# Special Notices for Hospital Sunday, 1889 A Year's Work in the Hospitals and Medical Charities of London

**Published:** 1889-06-15

**Authors:** 


### Special Notices as to Hospital Sunday, 1889.

### FOR THE CLERGY, MINISTERS OF RELIGION, AND OTHERS.

#### The Laity to the Rescue.

Until quite recent years the clergy and ministers of religion have been allowed by the laity to bear the whole heat and burden of the Hospital Sunday work. In 1886 a new departure was made by the institution of special meetings, supported by articles in the leading daily papers, day by day, throughout the week immediately preceding Hospital Sunday. These proceedings afforded most useful moral support to the clergy and ministers, who were stimulated to greater exertions, which resulted in the addition of several thousands of pounds to the collections. This year the Lord Mayor has organissd and presided at four great meetings in various parts of the metropolis in support of the hospitals ; and the council of the Hospital Sunday Fund have determined to confine their efforts to

#### One Great Meeting

On Wednesday, June 19, at 3 p.m., to be held in the Egyptian Hall of the Mansion House, the Lord Mayor, the Right Hon. Jas. Whitehead, in the chair. Invitations have been issued to the clergy, ministers of religion, churchwardens, and deacons, each ticket admitting three persons. In the event of others being required, they should be applied for immediately to the secretary of the Hospital Sunday Fund, Mansion House, E.C. The great feature of the meeting will be an address from the present president of the Royal College of Physicians.

#### Sir Andrew Clark, Bart., M.D., F.R.S., will be the principal speaker, and his wide knowledge of the subject, his eminence in the medical profession, and his proved eloquence as an orator, must combine to render his address not only interesting, but memorable in the history of the Hospital Sunday Fund movement.

The clergy and ministers of religion are, however, not to have it all their own way with the collections this year, or the proposal of Mr. Alfred Cohen to attempt to tap the sources of wealth in the City of London will this year be put to the test of practice.

#### A New Departure.

Mr. Cohen is desirous that the members of each of the great centres of commerce and business within the City, many of whom live outside of London, should have the claims of the hospitals brought specially under their notice by one or two of their number. It is proposed to circulate lists in each of these centres—to wit, the Stock Exchange, the Baltic, the Corn Exchange, Lloyds, the banking firms, and elsewhere. It will be interesting to note how far the laymen are able to compete with the clergy as collectors for the hospitals of London. Will the hundreds of members of each exchange, representing as they do wealth, influence, and prosperity, give of their abundance, and so produce larger collections than those forthcoming from any congregation in the metropolis ? We shall see. In any case, the clergy and ministers of religion will, we know, cordially recognise the kindly co-operation of the laity, and welcome it as one of the best signs of the times.

#### Free Copies of the Special Number.

Free copies of the Special Number of The Hospital may be had on personal written application at our offices, 139, Salisbury-court, E.C., by any clergyman, minister of religion, or churchwarden, who may require them for distribution amongst the members of their congregations. Although we are printing a special edition of 20,000 copies, experience shows that the demand is likely to be so great as to make it desirable that applications shall be made without delay.

#### Sermons and Subjects.

It is requested that an abstract of the special sermons, or at any rate the subject-matter of any such discourses, which may be preached in aid of the hospitals, at any of the services on Hospital Sunday, June 23, may be sent to the Editor of The Hospital, The Lodge, Porchester-square, W., not later than Tuesday evening, June 18, to enable them to appear in our next week's issue.

#### Donations and Cheques.

Any reader who desires to give to the Hospital Sunday Fund direct should make his cheque payable to Mr. Henry N. Custance, crossing it to the Bank of England, and sending it to him, as Secretary, at The Mansion House, E.C.

